# Identification of Emotional Spectrums of Patients Taking an Erectile Dysfunction Medication: Ontology-Based Emotion Analysis of Patient Medication Reviews on Social Media

**DOI:** 10.2196/50152

**Published:** 2023-11-29

**Authors:** Youran Noh, Maryanne Kim, Song Hee Hong

**Affiliations:** 1 College of Pharmacy Seoul National University Seoul Republic of Korea; 2 Research Institute of Pharmaceutical Sciences Seoul National University Seoul Republic of Korea

**Keywords:** erectile dysfunction, PDE5 inhibitor, social media, emotion analysis, sentiment analysis, emotions, patient medication experience, tailored patient medication, patient-centered care, men's health, medications, drugs

## Abstract

**Background:**

Patient medication reviews on social networking sites provide valuable insights into the experiences and sentiments of individuals taking specific medications. Understanding the emotional spectrum expressed by patients can shed light on their overall satisfaction with medication treatment. This study aims to explore the emotions expressed by patients taking phosphodiesterase type 5 (PDE5) inhibitors and their impact on sentiment.

**Objective:**

This study aimed to (1) identify the distribution of 6 Parrot emotions in patient medication reviews across different patient characteristics and PDE5 inhibitors, (2) determine the relative impact of each emotion on the overall sentiment derived from the language expressed in each patient medication review while controlling for different patient characteristics and PDE5 inhibitors, and (3) assess the predictive power of the overall sentiment in explaining patient satisfaction with medication treatment.

**Methods:**

A data set of patient medication reviews for sildenafil, vardenafil, and tadalafil was collected from 3 popular social networking sites such as WebMD, Ask-a-Patient, and Drugs.com. The Parrot emotion model, which categorizes emotions into 6 primary classes (surprise, anger, love, joy, sadness, and fear), was used to analyze the emotional content of the reviews. Logistic regression and sentiment analysis techniques were used to examine the distribution of emotions across different patient characteristics and PDE5 inhibitors and to quantify their contribution to sentiment.

**Results:**

The analysis included 3070 patient medication reviews. The most prevalent emotions expressed were joy and sadness, with joy being the most prevalent among positive emotions and sadness being the most prevalent among negative emotions. Emotion distributions varied across patient characteristics and PDE5 inhibitors. Regression analysis revealed that joy had the strongest positive impact on sentiment, while sadness had the most negative impact. The sentiment score derived from patient reviews significantly predicted patient satisfaction with medication treatment, explaining 19% of the variance (increase in *R*^2^) when controlling for patient characteristics and PDE5 inhibitors.

**Conclusions:**

This study provides valuable insights into the emotional experiences of patients taking PDE5 inhibitors. The findings highlight the importance of emotions in shaping patient sentiment and satisfaction with medication treatment. Understanding these emotional dynamics can aid health care providers in better addressing patient needs and improving overall patient care.

## Introduction

Erectile dysfunction (ED) is often accompanied by a wide range of emotions, including fear, anxiety, stress, and a negative mindset [1-3]. These emotional states can further worsen the condition. The introduction of phosphodiesterase type 5 (PDE5) inhibitors, such as sildenafil, vardenafil, and tadalafil, has revolutionized the treatment of ED [4]. These medications enhance erectile function by improving blood flow to the erectile tissue. However, apart from their physiological effects, PDE5 inhibitors may also have a significant impact on the emotional well-being of patients.

The emotional experience of patients with ED is influenced by various factors, including treatment efficacy [5], side effects [6], and individual expectations [7-9]. Different PDE5 inhibitors may have distinct effects on patients’ emotional states, leading to variations in drug preferences and engagement with drug therapy. Recognizing and addressing these patient emotions are crucial for delivering patient-centered care, promoting positive patient experiences, and ultimately improving medication treatment outcomes.

In recent decades, the science of emotion has undergone a revolution, leading to a paradigm shift in decision theories. Emotions have been recognized as playing a crucial role in treatment decision-making [10,11], shaping treatment preferences [12], treatment satisfaction [13,14], and patient-provider communication [15-17]. They interact with psychosocial factors [18] and are influenced by peer experiences and social media [19]. One study reported that patients’ emotional well-being can be negatively affected by changes in antiepileptic drug regimens [20]. However, little is known about the emotional experiences of patients taking PDE5 inhibitors.

There are significant barriers to identifying patient emotional experiences, including limited time during clinic visits [21,22] and the sensitive nature of ED. Patients visiting clinics often feel reluctant to express their emotional experiences due to time constraints. This reluctance is even more pronounced when it comes to discussing ED, which is a private matter. Patients taking PDE5 inhibitors may prefer to express their experiences on social media platforms that offer privacy and anonymity.

Various social media platforms have emerged as active forums for patients to share their medication experiences. Medication rating websites, such as WebMD, Ask-a-Patient, and Drugs.com, collect unstructured text comments from patients regarding their medication experiences. The anonymity provided by the digital space encourages patients with ED, who may fear stigma when discussing their emotional issues with doctors, to express their feelings more openly [23,24]. This helps capture the full spectrum of patient emotions expressed in their own words without embellishment or censorship [25].

This study aims to explore the spectrum of emotions expressed by patients taking PDE5 inhibitors by analyzing their patient medication reviews on social networking sites (SNSs). The specific objectives of this study are to (1) identify the distribution of 6 Parrot emotions (surprise, anger, love, joy, sadness, and fear) in patient medication reviews across different patient characteristics (such as age and duration of medication use) and PDE5 inhibitors (sildenafil, vardenafil, and tadalafil), (2) determine the relative impact of each emotion on the overall sentiment derived from the language expressed in each patient medication review while controlling for different patient characteristics and PDE5 inhibitors, and (3) assess the predictive power of the overall sentiment in explaining patient satisfaction with medication treatment.

Through these specific aims, the study intends to gain insights into the emotional experiences of patients taking PDE5 inhibitors and understand how these emotions contribute to their overall sentiment and satisfaction with medication treatment.

## Methods

### Data Structure and Parrot Emotion Ontology for Social Data

#### Social Media Data Source

Three websites were used as sources for collecting data on patient reviews of PDE5 inhibitors: WebMD [26], Ask-a-Patient [27], and Drugs.com [28]. These websites are popular social network platforms where individuals can share their experiences with medications. WebMD and Ask-a-Patient allow users to share medication reviews along with reviewer characteristics such as age, gender, and duration of medication use. Ask-a-Patient also collects information on drug dosage and includes a separate section for patients to comment on side effects. In contrast, Drugs.com does not collect reviewer characteristics other than the duration of medication use. All 3 websites allow patients to express their medication experiences through written reviews. Additionally, users can rate their medication on a scale of 1 to 5 (WebMD and Ask-a-Patient) or 1 to 10 (Drugs.com) for effectiveness, side effects, ease of use, satisfaction, and other aspects.

#### Emotion Ontology

This study adopted the Parrot emotion model [29], which has been previously used for emotion analysis of social media data [30,31]. Parrot’s model classifies emotions into 3 levels: primary, secondary, and tertiary. The primary emotion class consists of 6 emotions such as surprise, anger, love, joy, sadness, and fear, each of which further branches out into secondary and tertiary classes.

To enhance the ontology, synonyms for words contained in each tertiary emotion were added using Protégé (version 5.5; Stanford University), a widely used ontology editor. The synonyms were first identified from web-based dictionaries such as Merriam-Webster and Thesaurus.com, and additional slang expressions were collected through web searches, as proposed in previous studies [32,33]. This process ensured that newly developed words since the inception of the Parrot model were incorporated into our ontology. The final ontology consisted of 167 subclasses and 3167 synonyms. The reliability and consistency of the concepts in the final ontology were confirmed through Pellet reasoning tests.

### Data Collection and Preprocessing

**Figure 1:**
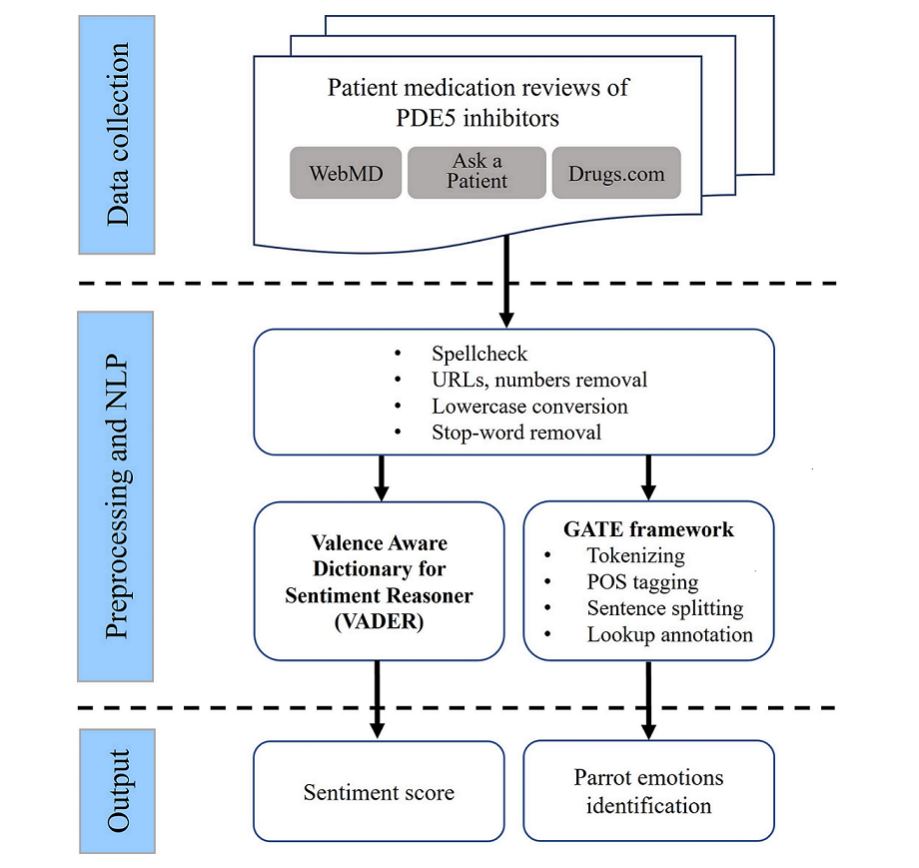
Overview of workflow for preparation of data and analyses. GATE: General Architecture for Text Engineering; NLP: natural language processing; PDE5: phosphodiesterase type 5; POS: part-of-speech.

Using Python (version 3.8.12; Python Software Foundation), we searched patient medication reviews of sildenafil, vardenafil, and tadalafil posted between 2001 and 2022 on WebMD, Ask-a-Patient, and Drugs.com. Reviews for both brand name and generic drugs were classified into the same drug category. Side effect comments from the separate section of the Ask-a-Patient website were merged with the overall medication review comments. For reviews collected from Drugs.com, patient age (when available) was extracted from each comment, since Drugs.com does not have a dedicated section for patient age. Spellcheck was applied to all reviews using *Textblob*, a Python library. To minimize possible noise in the medication reviews, text preprocessing techniques were applied, such as removing web links and numbers, converting text to lowercase, and removing stop words (Figure 1).

### Sentiment Analysis of Patient Medication Reviews

VADER (Valence Aware Dictionary for Sentiment Reasoner), a widely recognized sentiment analysis tool, was used to generate a sentiment score for each patient medication review. VADER is specifically designed for sentiment analysis of social media posts, which often contain slang and informal language [34]. VADER provides a standardized sentiment score ranging from –1 for the most negative sentiment to +1 for the most positive sentiment.

### Emotion Analysis of Patient Medication Reviews

The final ontology was integrated into the General Architecture for Text Engineering 9.0 natural language processing tool to annotate emotional terms in medication reviews. The ontology was incorporated into the OntoRoot Gazetteer, a component of the General Architecture for Text Engineering software. A pipeline of natural language processing tools, including an English tokenizer, part-of-speech tagger, sentence splitter, and morphological analyzer, was used to annotate emotional terms in the medication reviews based on the ontology. The Java Annotation Patterns Engine transducer was then applied to the annotated corpus to extract the second-level classes of the emotion ontology by matching them with their corresponding tertiary-level emotion classes or synonyms. These extracted emotional terms were subsequently grouped into Parrot top-tier 6 primary emotion classes. The Java Annotation Patterns Engine transducer consists of a set of modifiable rules comprising phrases that determine the actions executed during the annotation process.

### Statistical Analysis

The prevalence of emotional spectrums detected in the patient medication reviews was assessed using chi-square tests to analyze independent distributions across patient characteristics such as medication name, patient’s age, and duration of medication use. Logistic regression was performed to examine the prevalence of each emotion across different patient characteristics.

The predictive power of the sentiment score in explaining patient satisfaction with medication therapy was assessed by including the sentiment score as a variable in a regression model. The increase in *R*^2^ when the sentiment score was included in the model was used to evaluate the extent to which the sentiment score explains patient satisfaction while controlling for PDE5 inhibitors, patient demographics, and the year of the review.

To determine the impact of each emotion on medication therapy sentiment, regression analysis was conducted, again controlling for different patient characteristics. All statistical analyses were performed using R statistical software (version 4.1.1; R Foundation for Statistical Computing), and *P* values less than .05 were considered statistically significant.

### Ethical Considerations

The data collected for this study are publicly available from SNSs; accessible to all internet users; and do not include any personally identifiable information such as names, dates of birth, and addresses. We ensured data security and user privacy and obtained ethical approval from the Seoul National University institutional review board for this study (IRB 2211/002-031).

## Results

### Patient Characteristics of PDE5 Inhibitor Medication Reviewers

A total of 3193 PDE5 inhibitor medication reviews were initially collected from 3 websites, with 1022 for sildenafil, 419 for vardenafil, and 1752 for tadalafil. After excluding duplicate and nontextual posts, the final data set consisted of 3070 reviews, including 973 for sildenafil, 406 for vardenafil, and 1691 for tadalafil (Table 1). The greatest portion of the reviews (371/973, 38.1% for sildenafil; 165/406, 40.6% for vardenafil; and 716/1691, 42.3% for tadalafil) were provided by adults aged 45-64 years. Approximately one-third (283/973, 29.1% for sildenafil; 110/406, 27.1% for vardenafil; 608/1691, 36% for tadalafil) of the reviews were written by individuals who had been taking the drug for less than a month. Over the 2-decade period, most reviews were posted between 2010 and 2019. Notably, from 2010 to 2014, the number of posts increased for sildenafil (from 265 to 341) but decreased by over 50% (from 186 to 90) for vardenafil. This decline may be attributed to GlaxoSmithKline’s decision to halt commercial advertising for Levitra on television in 2011.

**Table 1:** Distribution of characteristics in patient medication reviews of phosphodiesterase type 5 inhibitors.

Characteristics		Sildenafil (n=973)	Vardenafil (n=406)	Tadalafil (n=1691)	Total (n=3070)
**Age (years), n (%)**					
	<44	169 (17.4)	54 (13.3)	324 (19.2)	547 (17.8)
	45-64	371 (38.1)	165 (40.6)	716 (42.3)	1252 (40.8)
	≥65	99 (10.2)	68 (16.7)	194 (11.5)	361 (11.8)
	N/A^a^	334 (34.3)	119 (29.3)	457 (27)	910 (29.6)
**Duration of medication, n (%)**					
	Short-term use^b^	283 (29.1)	110 (27.1)	608 (36)	1001 (32.6)
	Midterm use^c^	216 (22.2)	88 (21.7)	372 (22)	676 (22)
	Long-term use^d^	252 (25.9)	92 (22.7)	321 (19)	665 (21.7)
	N/A	222 (22.8)	116 (28.6)	390 (23.1)	728 (23.7)
**Rating year, n (%)**					
	2001-2009	252 (33.2)	120 (15.7)	387 (50.5)	759 (24.7)
	2010-2019	606 (30)	276 (13.7)	1138 (56.3)	2020 (65.8)
	2020-2022	115 (39.5)	10 (3.4)	166 (57)	291 (9.5)
Self-reported medication treatment satisfaction scores, mean (SD)^e^		4.01 (1.30)	3.97 (1.33)	3.83 (1.43)	3.90 (1.38)
**Number of characters and sentences of medication reviews, mean (SD)**					
	Characters	364.55 (304.50)	309.40 (268.79)	405.98 (307.24)	380.08 (303.30)
	Sentences	5.26 (4.00)	4.54 (3.86)	5.76 (4.08)	5.44 (4.05)

^a^N/A: not available.

^b^Short-term use means less than 1 month.

^c^Midterm use means from 1 month to less than 1 year.

^d^Long-term use means more than 1 year.

^e^The mean satisfaction score of patients regarding their medication was quantified through a 5-point Likert scale and expressed as a mean value. For the purpose of consistency and comparability, the scores obtained from Drugs.com were standardized to a 5-point Likert scale by converting the original criteria, which were based on a 10-point Likert scale.

### Emotional Spectrums by Patient Characteristics and Different PDE5 Inhibitors

Among the 6 Parrot emotions, joy was the most prevalent, appearing in 59.61% (1830/3070) of the reviews or comments (Table 2). It had the highest prevalence among the positive emotions (joy, love, and surprise) and was at least 8% (536/1002, 53.49% for joy vs 448/1002, 44.71% for sadness) more prevalent than the most common negative emotion, sadness. The prevalence of joy was similar across different PDE5 inhibitors ranging from 58.07% (565/973) to 61.08% (248/406). However, sadness appeared twice as often in tadalafil compared to sildenafil and vardenafil. Younger adults expressed both joy and sadness more frequently than those aged 65 years or older. Regarding the duration of medication use, individuals who had just started taking the medication (less than 1 month) expressed joy less frequently but expressed sadness more frequently compared to those with a duration of medication use between 1 month and 1 year, as well as those with over 1 year of medication use.

The logistic regression analysis (Table 3) revealed that the likelihood of expressing joy was not statistically different between tadalafil and sildenafil (odds ratio [OR] 1.12, 95% CI 0.95-1.32). However, the likelihood of expressing surprise was lower in tadalafil than in sildenafil (OR 0.79, 95% CI 0.64-0.97). Moreover, the likelihood of expressing sadness (OR 3.85, 95% CI 3.18-4.68) was significantly higher in tadalafil compared to sildenafil. Conversely, the likelihood of expressing fear (OR 0.71, 95% CI 0.64-0.97), a stronger negative emotion than sadness, was significantly lower in tadalafil than in sildenafil. The age groups also exhibited significant differences in the likelihood of expressing each Parrot emotion. The younger age groups had significantly higher likelihoods of expressing joy and surprise (joy: OR 1.69, 95% CI 1.29-2.22; surprise: OR 2.00, 95% CI 1.36-3.01) compared to the reference age group (65 years or older). The younger age groups were also more likely to express sadness (OR 1.86, 95% CI 1.37-2.54). Regarding the duration of medication use, individuals who had taken the drug for more than 1 year were more likely to express joy than those who had been taking the drug for less than 1 month (OR 1.41, 95% CI 1.15-1.73). The likelihood of expressing sadness tended to decrease as the duration of medication use increased; the OR for expressing sadness was 0.49 (95% CI 0.55-0.59) for midterm use and 0.61 (95% CI 0.48-0.75) for long-term use, both compared to the reference short-term use.

**Table 2:** Emotional spectrums identified from patient medication reviews by phosphodiesterase type 5 inhibitor, age, and duration of medication.

Characteristics^a^		Primary emotions, % (SE)						*P* value
		Joy	Love	Surprise	Sadness	Fear	Anger	
**Drug**								<.001
	Sildenafil	58.07 (0.016)	18.4 (0.012)	19.42 (0.013)	18.29 (0.012)	17.47 (0.012)	4.93 (0.007)	
	Vardenafil	61.08 (0.024)	17.73 (0.019)	15.27 (0.018)	18.97 (0.019)	9.36 (0.014)	5.17 (0.011)	
	Tadalafil	60.14 (0.012)	19.52 (0.010)	16.14 (0.009)	46.9 (0.012)	14.02 (0.008)	6.09 (0.006)	
**Age (years)**								.001
	<44	61.61 (0.021)	19.56 (0.017)	20.29 (0.017)	41.32 (0.021)	22.67 (0.018)	6.58 (0.011)	
	45-64	60.22 (0.014)	19.25 (0.011)	15.65 (0.010)	40.97 (0.014)	13.5 (0.010)	5.75 (0.007)	
	≥65	50.14 (0.026)	18.84 (0.021)	10.8 (0.016)	26.04 (0.023)	10.25 (0.016)	6.09 (0.013)	
**Duration of medication**								<.001
	Short-term use^b^	53.49 (0.016)	17.96 (0.012)	16.77 (0.012)	44.71 (0.016)	18.06 (0.012)	6.59 (0.008)	
	Midterm use^c^	61.33 (0.019)	18.67 (0.015)	15.26 (0.014)	28.44 (0.017)	13.63 (0.013)	4.89 (0.008)	
	Long-term use^d^	60.6 (0.019)	19.4 (0.015)	13.68 (0.013)	30.23 (0.018)	9.77 (0.012)	5.86 (0.009)	

^a^For each characteristic, the frequency of primary emotion is provided.

^b^Short-term use means less than 1 month.

^c^Midterm use means from 1 month to less than 1 year.

^d^Long-term use means more than 1 year.

**Table 3:** Associative primary emotions for phosphodiesterase type 5 inhibitor, age, and duration of medication.

Characteristics		Positive emotions, OR^a^ (95% CI)			Negative emotions, OR (95% CI)		
		Joy	Love	Surprise	Sadness	Fear	Anger
**Drug**							
	Sildenafil	Reference	Reference	Reference	Reference	Reference	Reference
	Vardenafil	1.15 (0.91-0.47)	0.95 (0.70-1.28)	0.75 (0.54-1.03)	1.06 (0.78-1.42)	0.49 (0.33-0.71)	1.06 (0.61-1.77)
	Tadalafil	1.12 (0.95-1.32)	1.07 (0.88-1.32)	0.79 (0.64-0.97)	3.85 (3.18-4.68)	0.71 (0.57-0.89)	1.22 (0.86-1.76)
**Age (years)**							
	< 44	1.69 (1.29-2.22)	1.05 (0.75-1.49)	2.00 (1.36-3.01)	1.86 (1.37-2.54)	2.25 (1.53-3.40)	1.07 (0.62-1.89)
	45-64	1.54 (1.22-1.96)	1.03 (0.76-1.39)	1.49 (1.05-2.18)	1.98 (1.51-2.62)	1.28 (0.88-1.89)	0.93 (0.58-1.57)
	≥65	Reference	Reference	Reference	Reference	Reference	Reference
**Duration of medication**							
	Short-term use^b^	Reference	Reference	Reference	Reference	Reference	Reference
	Midterm use^c^	1.40 (1.15-1.72)	1.06 (0.82-1.36)	0.90 (0.69-1.18)	0.49 (0.39-0.61)	0.72 (0.55-0.95)	0.74 (0.47-1.13)
	Long-term use^d^	1.41 (1.15-1.73)	1.11 (0.86-1.43)	0.82 (0.62-1.08)	0.61 (0.48-0.75)	0.51 (0.37-0.69)	0.91 (0.60-1.37)

^a^OR: odds ratio.

^b^Short-term use means less than 1 month.

^c^Midterm use means from one month to less than 1 year.

^d^Long-term use means more than 1 year.

### Effect of Each Parrot Emotion on Sentiment

All of the Parrot emotions significantly predicted the sentiment expressed in the medication reviews (Table 4). Controlling for PDE5 inhibitors, patient demographics, and the year of the review, positive emotions such as love, surprise, and joy had a positive impact on the sentiment (β=.249, *P*<.001 for love; β=.287, *P*<.001 for surprise; and β=.467, *P≤*.001 for joy), while negative emotions (sadness, fear, and anger) had a negative impact (β=–.433, *P≤*.001 for sadness; β=–.139, *P*≤.001 for fear; and β=–.114, *P*=.005 for anger). Among the positive emotions, joy had the strongest positive influence on the sentiment, while sadness had the most pronounced negative effect.

**Table 4:** Exploratory multiple regression analysis on the Parrot emotions contributing to the medication review sentiment controlling for patient characteristics and phosphodiesterase type 5 inhibitors^a^.

Variables		β	SE	*P* value
Intercept		–.16	0.046	<.001
**Parrot primary emotions**				
	Sadness	–.433	0.022	<.001
	Fear	–.139	0.027	<.001
	Anger	–.114	0.040	.005
	Love	.249	0.024	<.001
	Surprise	.287	0.025	<.001
	Joy	.467	0.019	<.001
*R* ^2^		.37		

^a^Regarding potential confounders controlled in the regression model, tadalafil reviews showed significantly decreased medication review sentiment (β=–.067; *P*=.002; reference=sildenafil). Age did not significantly explain the sentiment. The patients using phosphodiesterase type 5 inhibitors for more than 1 month showed significantly greater increases in the sentiment (β=.155, *P*<.001 for midterm use; β=.141, *P*<.001 for long-term use; reference=short-term use). Recent years’ reviews of PDE5 inhibitors showed a slight but significant increase in sentiment compared to preceding years (β=.005; *P*=.04).

### Predictive Power of Sentiment on Satisfaction With Medication Treatment

When the regression model was run with the inclusion of the sentiment variable (Table 5), the *R*^2^ value increased from 0.06928 to 0.259. This increase of 0.18972 in *R*^2^ indicates that 18.97% of the variance in patient satisfaction with medication treatment is explained by the medication review sentiment, after controlling for PDE5 inhibitors, patient demographics, and the year of the review.

These findings highlight the significant role of emotions in shaping the sentiment expressed in PDE5 inhibitor medication reviews. Understanding the impact of emotions and sentiment on patient satisfaction can inform health care providers in tailoring interventions and communication strategies to enhance positive emotions, manage negative emotions, and ultimately improve overall sentiment and patient satisfaction.

**Table 5:** Exploratory multiple regression models examining the extent of medication review sentiment contributing to the medication treatment satisfaction score.

Characteristics		Model 1^a^			Model 2^b^		
		β	SE	*P* value	β	SE	*P* value
Intercept		3.04	0.11	<.001	2.92	0.12	<.001
Medication review sentiment		.97	0.04	<.001	—	—	—
**Drug** **(n=2946)**							
	Sildenafil (n=926)	Reference	Reference	Reference	Reference	Reference	Reference
	Vardenafil (n=387)	–.07	0.07	.35	.01	0.08	.91
	Tadalafil (n=1633)	.04	0.05	.40	–.13	0.06	.02
**Age (years)**							
	<44	.32	0.08	<.001	.35	0.09	<.001
	45-64	.26	0.07	<.001	.29	0.08	<.001
	≥65	Reference	Reference	Reference	Reference	Reference	Reference
**Duration of medication**							
	Short-term use^c^	Reference	Reference	Reference	Reference	Reference	Reference
	Midterm use^d^	.43	0.06	<.001	.69	0.07	<.001
	Long-term use^e^	.57	0.06	<.001	.81	0.07	<.001
Rating year		.01	0.01	.03	.02	0.01	<.001
*R*^2^ (adjusted)		0.26			0.07		

^a^Model 1 is the regression model adjusted including medication sentiment.

^b^Model 2 is the regression model adjusted except for medication sentiment.

^c^Short-term use means less than 1 month.

^d^Midterm use means from 1 month to less than 1 year.

^e^Long-term use means more than 1 year.

## Discussion

### Principal Findings

The emotion ontology developed in this study, based on Parrot emotion model, revealed a distinct distribution of the 6 primary Parrot emotions across different patient characteristics, including the specific PDE5 inhibitors used, patient ages, and duration of medication use. While the emotion of joy appeared at similar frequencies across various PDE5 inhibitors, sadness was more frequently expressed for tadalafil compared to sildenafil. This higher prevalence of sadness for tadalafil may be attributed to its longer half-life, which can lead to prolonged side effects [35-37] compared to the shorter half-lives of sildenafil and vardenafil [38]. Furthermore, the higher frequency of fear reported for sildenafil could be associated with the occurrence of severe adverse reactions such as abnormal vision, as indicated in previous studies [39,40]. Additionally, the increased frequency of surprise expressed for sildenafil may align with its reputation for faster and more powerful effects, contrasting with tadalafil’s emphasis on romance [41].

Among different age groups, the emotions of joy and surprise were significantly more prevalent in younger individuals, while sadness and fear were also more frequently expressed in these age groups. The higher rates of joy and surprise in younger individuals may be attributed to their heightened responsiveness to sexual stimulation compared to older men [42], who may require more stimulation to achieve an erection [43]. Conversely, the higher rates of sadness and fear in younger age groups may be associated with the more frequent abuse and misuse of PDE5 inhibitors for sexual enhancement, leading to an increased occurrence of adverse events in younger men [44].

Furthermore, patients who had been using PDE5 inhibitors for a longer duration were more likely to express joy and less likely to express sadness. Previous research has shown that patients with ED are prone to discontinuing PDE5 inhibitor use within 2-3 years [45]. The finding that patients with a longer duration of medication use express more joy and less sadness is not surprising, as it indicates that patients who continue taking the medication for an extended period are likely to experience positive treatment outcomes without significant adverse reactions. Recognizing the emotional trajectory throughout the course of medication use can provide valuable insights for interventions aimed at improving treatment adherence and patient satisfaction.

Our results also demonstrated that each of the 6 emotions made a significant contribution to sentiment. Positive emotions such as joy, love, and surprise had a positive effect on sentiment, while negative emotions such as sadness, fear, and anger had a negative effect. Emotions, which encompass intense feelings in response to stimuli, are inherently interconnected with sentiment, representing the overall attitude that individuals express toward something [46]. In the context of our research, emotions can influence the sentiment expressed in patient reviews regarding their use of PDE5 inhibitors. Positive emotions can contribute to a favorable sentiment toward the drug, leading to improved medication adherence [47] and treatment outcomes [48]. Conversely, negative emotions can contribute to a negative sentiment, potentially indicating dissatisfaction. In fact, our study revealed that the sentiment uncovered from patient medication reviews explained a significant portion of the variance in patient satisfaction with medication treatment (increase in *R*^2^=18.97%). Dissatisfaction with medication treatment has been associated with nonadherence, off-label use, and even the consumption of illegal substances [49,50].

Understanding the relationship between emotions and sentiment is invaluable in the health care context. By analyzing and addressing the underlying emotions that contribute to sentiment, health care providers can tailor their interventions and communication strategies to enhance positive emotions, manage negative emotions, and ultimately improve overall sentiment and patient satisfaction. This knowledge can guide health care professionals in delivering patient-centered care that recognizes and addresses the emotional experiences of patients using PDE5 inhibitors and potentially extend to other areas of health care as well.

### Practice Implication

The findings of this study have important practice implications for health care providers treating patients with ED who are taking different PDE5 inhibitors. The emotional spectrums identified in this study highlight the need for physicians to recognize and understand the varying emotional states of their patients based on individual characteristics. Given the private nature of ED, patients may not feel comfortable openly communicating their emotions with their physicians. Therefore, it is crucial for health care providers to have access to the emotional needs of their patients from alternative sources such as web-based patient medication reviews.

By integrating sentiment analysis and ontology-based emotion identification into clinical practice, health care providers can better understand their patients’ experiences, tailor treatment approaches, and improve patient satisfaction. This comprehensive understanding of patient emotions and sentiments can lead to more effective communication, enhanced patient-provider relationships, and ultimately, improved health care outcomes for patients with ED.

### Limitations

There are several limitations to consider in this study. First, the application of the Parrot emotion model directly for social media data may have limitations in detecting the complete emotional spectrums present in patient medication reviews, as the model may not encompass all the terms and expressions commonly used in social media. Although we supplemented the emotion ontology with web-based dictionaries and incorporated slang used on the web, it is important to acknowledge that our emotion ontology may not capture every possible emotional expression in its entirety.

Second, measuring sentiment accurately remains a challenge in the field of machine learning, particularly when it comes to identifying sarcasm and irony [51-53]. These nuanced forms of expression can significantly impact the interpretation of emotional responses in natural language. Therefore, developing reliable methods for detecting and interpreting such nuances in sentiment analysis is an ongoing research question that needs further exploration.

Another limitation is the potential presence of selection bias in the patient medication reviews voluntarily posted on SNSs. It is possible that the reviews tend to exhibit a skewed distribution, with a greater representation of extremely positive or negative medication experiences. Furthermore, there may be a bias toward specific demographic groups, such as younger individuals and those with higher socioeconomic status, who are more likely to engage in sharing their experiences on SNSs. This could limit the generalizability of the findings to a broader population of patients with ED.

Despite these limitations, this study provides valuable insights into the emotional experiences and sentiments expressed by patients taking PDE5 inhibitors for ED. Future research should aim to address these limitations by refining emotion models, improving sentiment analysis techniques, and ensuring a more diverse and representative sample of patient medication reviews.

### Conclusions

The unique spectrum of Parrot emotions, combined with varying sentiments identified from medication reviews on SNSs, highlights the importance of considering patient characteristics in understanding and addressing patient emotions. This finding holds significant implications for the design of tailored patient medication counseling, where the focus can be centered on addressing and acknowledging the specific emotional needs of individual patients.

This study underscores the significance of incorporating patient emotions into the therapeutic process, particularly for individuals with ED who may be hesitant to openly discuss their emotional experiences with health care providers due to the sensitive nature of the condition. By acknowledging and addressing these emotions, health care professionals can foster a more supportive and patient-centered approach to medication counseling, ultimately enhancing patient satisfaction and treatment adherence.

Moving forward, future research should continue to explore and refine the understanding of patient emotions within the context of medication experiences. By further investigating the interplay between emotions, sentiments, and patient characteristics, we can advance the development of comprehensive strategies that optimize patient-centered care in the management of ED and potentially extend to other health care domains as well.

## Appendix

Multimedia Appendix 1 Medication reviews of PDE5 inhibitors collected from SNS platforms.

## Abbreviations

ED: erectile dysfunction
OR: odds ratio
PDE5: phosphodiesterase type 5
SNS: social networking site
VADER: Valence Aware Dictionary for Sentiment Reasoner

## Data Availability

All data generated or analyzed during this study are included in this published article as Multimedia Appendix 1.
